# An image of multiple bullous lesions in a patient on hemodialysis

**DOI:** 10.1590/2175-8239-JBN-2026-0077en

**Published:** 2026-06-22

**Authors:** Ana Claudia Siqueira Marques, Rafael Fantelli Stelini, Rodrigo Bueno de Oliveira

**Affiliations:** 1 Universidade Estadual de Campinas, Faculdade de Ciências Médicas, Departamento de Medicina Interna, Divisão de Nefrologia, Campinas, SP, Brazil.; 2 Universidade Estadual de Campinas, Faculdade de Ciências Médicas, Departamento de Patologia, Campinas, SP, Brazil.; 3 Centrolab, Campinas, SP, Brazil.; 4 Universidade Estadual de Campinas, Faculdade de Ciências Médicas, Laboratório para o Estudo Mineral e Ósseo em Nefrologia, Campinas, SP, Brazil.

A 27-year-old woman with diabetes, epilepsy, and ESKD undergoing hemodialysis presented with painful bullous lesions on the hands and face, which developed after treatment for a central venous catheter (CVC)-related fungal bloodstream infection. Toxic epidermal necrolysis (TEN), porphyria cutanea tarda, and iron-associated pseudoporphyria were considered^1,2^. There were no clinical or laboratory findings suggestive of autoimmune disease. Urinary and genetic tests were negative for porphyria. Histopathological examination of the skin revealed features consistent with porphyria and TEN. Improvement occurred after discontinuation of fluconazole and carbamazepine, photoprotection, removal of the CVC and adhesive dressings, initiation of prednisone, and phlebotomy to decrease iron overload (Figure 1).

**Figure 1:**
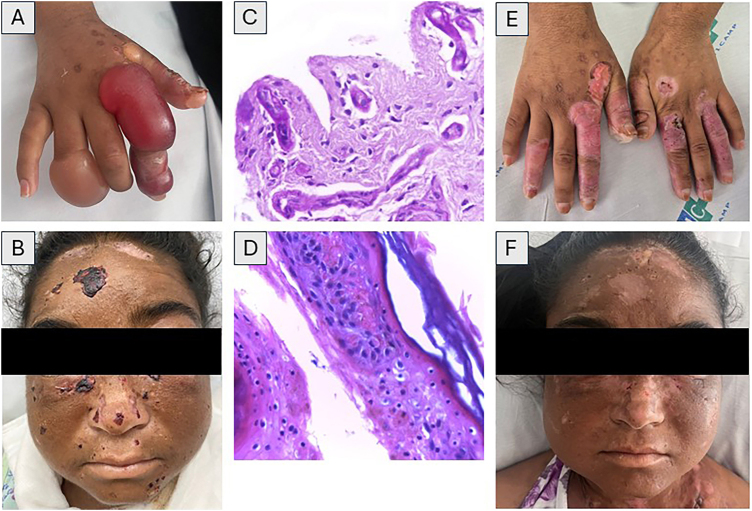
Multiple bullous lesions, histopathological examination of the skin, and remission of skin lesions after treatment. A and B, bullous lesions on the right hand and face before treatment; C, histopathological section of the dermis (“floor of the blisters”) showing absence of the epidermis, with festooning dermal papillae and mild thickening of superficial vessel walls, findings that may be compatible with porphyria or pseudoporphyria (periodic acid-Schiff, magnification of ×200); D, “top of the blister” evidencing epidermal necrosis segmental and of individual keratinocytes, findings that may correspond to toxic epidermal necrolysis (hematoxylin and eosin; magnification: ×200). Direct immunofluorescence revealed deposits of C3 (moderate), IgG (mild), and IgM (mild) in the vessel wall (not shown); E and F, remission of skin lesions on the hands and face, and the healing process.

## Data Availability

All data related to this publication are made available on reasonable request.
