# Supplementary material for: An image of multiple bullous lesions in a patient on hemodialysis
Source: J Bras Nefrol. 2026 Jun 22;48(3):e20260077. doi: 10.1590/2175-8239-JBN-2026-0077en (PMC13317795; doi:10.1590/2175-8239-JBN-2026-0077en)
Supplement: Translation

# Translation: Uma imagem de lesões bolhosas múltiplas em um paciente em hemodiálise

**DOI:** 10.1590/2175-8239-JBN-2026-0077pt

**Authors:** Ana Claudia Siqueira Marques, Rafael Fantelli Stelini, Rodrigo Bueno de Oliveira

**Affiliations:** 1 Universidade Estadual de Campinas, Faculdade de Ciências Médicas, Departamento de Medicina Interna, Divisão de Nefrologia, Campinas, SP, Brasil.; 2 Universidade Estadual de Campinas, Faculdade de Ciências Médicas, Departamento de Patologia, Campinas, SP, Brasil.; 3 Centrolab, Campinas, SP, Brasil.; 4 Universidade Estadual de Campinas, Faculdade de Ciências Médicas, Laboratório para o Estudo Mineral e Ósseo em Nefrologia, Campinas, SP, Brasil.

Uma mulher de 27 anos com diabetes, epilepsia e DRC em hemodiálise apresentou lesões bolhosas dolorosas nas mãos e no rosto, que se desenvolveram após o tratamento de uma infecção fún­gica da corrente sanguínea relacionada ao cateter venoso central (CVC). Foram consideradas necrólise epidérmica tóxica (NET), porfiria cutânea tardia e pseudoporfiria associada ao ferro^1,2^. Não havia achados clínicos ou laboratoriais sugestivos de doença autoimune. Os exames urinários e os testes genéticos foram negativos para porfiria. O exame histopatológico da pele revelou características descritas na porfiria e NET. Houve melhora após a suspensão do fluconazol e da carbamazepina, a fotoproteção, a remoção do CVC e dos curativos adesivos, o início do tratamento com prednisona e a flebotomia para reduzir a sobrecarga de ferro (Figura 1).

**Figura 1:** Lesões bolhosas múltiplas, exame histopatológico da pele e remissão das lesões cutâneas após tratamento. A e B, lesões bolhosas na mão direita e no rosto antes do tratamento; C, imagem histológica da derme (“assoalho da bolha”) mostrando ausência de epiderme, com papilas dérmicas festonadas e leve espessamento da parede dos vasos superficiais, achados que podem corresponder à porfiria ou pseudoporfiria (ácido periódico de Schiff; ×200); D, “teto da bolha” evidenciando necrose epidérmica, segmentar e de ceratinócitos individuais, achados que podem corresponder à necrólise epidérmica tóxica (hematoxilina e eosina; ×200). A imunofluorescência direta revelou depósitos de C3 (moderado), IgG (leve) e IgM (leve) na parede de vasos (não mostrado); E e F, remissão das lesões cutâneas nas mãos e no rosto e processo de cicatrização.

## Data Availability

Todos os dados relacionados a esta publicação estão disponíveis mediante solicitação razoável.
